# Maternal exposure to pesticides during pregnancy and risk for attention-deficit/hyperactivity disorder in offspring: A protocol for systematic review and meta-analysis

**DOI:** 10.1097/MD.0000000000026430

**Published:** 2021-07-02

**Authors:** Zhixian Zhu, Ying Wang, Shiming Tang, Huawei Tan, Cheng Liu, Liwei Cheng

**Affiliations:** a Mental Health Center; b Department of Gynaecology; c Office of Academic Research, Renmin Hospital of Wuhan University, Jiefang Road 238, Wuchang District, Wuhan, PR China.

**Keywords:** attention-deficit/hyperactivity disorder, pesticide, protocol, systematic review

## Abstract

**Background:**

Attention-deficit/hyperactivity disorder (ADHD) is the most common psychiatric disorder in childhood. Studies explored the association of maternal exposure to pesticides during pregnancy with a risk of offspring developing ADHD, but have reported inconclusive results. Here, we will perform a systematic review and meta-analysis of observational studies to assess a possible association between them.

**Methods:**

This study followed the Preferred Reporting Items for Systematic Reviews and Meta-Analyses Protocols. PubMed, Embase, Web of Science, the Cochrane Library, and the PsycINFO will be searched from inception to May 2021. Observational studies investigating the association of maternal exposure to pesticides during pregnancy with a risk of offspring developing ADHD will be considered. The Newcastle-Ottawa Scales or the scale of the Agency for Healthcare Research and Quality will be used to assess the methodological quality of the included studies according to their study design. A fixed or random-effect model will be used to synthesize data depend on the heterogeneity test. STATA version 12.0 will be used to conduct the meta-analysis.

**Results:**

This study will provide a high-quality evaluation of association between maternal exposure to pesticides during pregnancy and risk for ADHD in offspring.

**Conclusion:**

This study will present evidence on whether maternal exposure to pesticides during pregnancy is a risk factor for ADHD in offspring.

## 1 Introduction

Attention-deficit/hyperactivity disorder (ADHD) is a childhood-onset neurodevelopmental disorder characterized by developmentally inappropriate inattentiveness, hyperactivity, and increased impulsivity.^[1]^ As the most common psychiatric disorder in childhood, ADHD often persists into adulthood and is estimated to affect around 5% of children under 18 years of age and 2.5% of adults worldwide.^[2,3]^ ADHD is associated with a wide range of other mental health problems, including affective disorders, defiant and antisocial personality disorder, self-harm, and substance misuse. In addition, ADHD may seriously disrupt one's social life, leading to a series of negative outcomes, such as educational underachievement, unemployment, and criminality.^[3,4]^ While short-term medication-based treatments for ADHD have proven to be efficacious and cost-effective, the long-term effectiveness of these treatments, regarding remains uncertain on key educational, vocational, and social outcomes. Moreover, the effectiveness of non-pharmacological approaches to treatment has proven to be lower than expected.^[5]^ Therefore, primary prevention targeting at risk factors for ADHD is of great importance to the prevention and management of ADHD from a public health perspective.

Although the exact etiology remains unknown, ADHD is suggested to be a highly heritable disorder, with an estimated heritability of 70% to 80%.^[5,6]^ Environmental factors may also contribute to the development of ADHD, as is indicated by the increased prevalence of ADHD over the last few decades, during which genes are stable.^[7]^ Among numerous environmental factors, many prenatal risk factors, such as intrauterine exposure to tobacco, maternal stress, and obesity during pregnancy, have been demonstrated to be associated with an increased risk of ADHD.^[5]^ The identification of these prenatal risk factors calls for special attention and sufficient interventions for pregnant women.

Pesticides are a group of complex chemicals or biological agents used for destroying or mitigating pest,^[8]^ and are intensively used worldwide during the past century. Humans can be exposed to pesticides directly through occupational, agricultural, and/or household use of pesticides, or indirectly through the circulation and accumulation of pesticides in the food chain.^[9]^ Studies demonstrated that maternal exposure to pesticides during pregnancy is related to an increased risk of adverse reproductive outcomes and neurodevelopmental disorders in offspring.

In recent years, several studies focused on the potential effects of maternal exposure to pesticides during pregnancy on the ADHD risk in offspring; however, the results of these studies were inconclusive. Some studies found that maternal exposure to chlorpyrifos, pyrethroids, and p, p’-dichlorodiphenyldichloroethylene during pregnancy was associated with an increased ADHD risk in offspring,^[10–12]^ though a similar association was not confirmed in other studies.^[7,13–18]^ Therefore, the purpose of this study will be to explore whether there is an association between maternal exposure to pesticides during pregnancy with the ADHD risk in offspring.

## 2 Methods

### 2.1 Ethics and dissemination

Ethical approval is not applicable, since this study will not involve individual data.

### 2.2 Study registration

The systematic review and meta-analysis has been registered in INPLASY (registration number: INPLASY202150088, DOI: 10.37766/inplasy2021.5.0088). It will be reported in accordance with the Preferred Reporting Items for Systematic Reviews and Meta Analyses Protocols guidelines^[19]^ to this protocol.

### 2.3 Eligibility criteria

#### 2.3.1 Participants

Pregnant women and their offspring will be included.

#### 2.3.2 Intervention (exposure)

The exposure factor is pesticides, including herbicides, insecticides, fungicides, bactericides, rodenticides, fumigants, and any of their subtypes. The level of maternal household and agricultural exposure will be ascertained by measurements of maternal serum or unary biomarkers, or by estimation from previous pesticide application data.

#### 2.3.3 Comparison

Offspring participants whose mothers were not exposed or were only exposed to a relatively low dose of pesticides during pregnancy will be used as the control group.

#### 2.3.4 Outcomes

The outcome is the incidence of ADHD, which will be ascertained by the International Classification of Diseases or the Diagnostic and Statistical Manual of Mental Disorders, or their related diagnostic tools.

#### 2.3.5 Inclusion criteria

1. Observational studies, including cohort, case–control, and cross-sectional studies, will be included in this study.
2. These studies should have reported the relative risk (RR) or odds ratio (OR) and their corresponding 95% confidence interval (CI) of association of maternal exposure to pesticides during pregnancy with the ADHD risk in offspring, or have reported any original data to compute the aforementioned risk estimates and 95% CI.
3. If more than one study is generated from the same cohort, the study that reported the largest number of ADHD cases will be included.

#### 2.3.6 Exclusion criteria

Duplicate studies, non-human studies, case reports or case series studies, conference abstracts, reviews, comments, and letters will be excluded.

### 2.4 Information sources

Relevant studies will be identified by searching PubMed, Embase, Web of Science, the Cochrane Library, and the PsycINFO. All of the databases will be searched from inception to May 2021.

### 2.5 Search strategy

The main search terms will include “pregnancy,” “pesticide,” “organochlorines,” “organophosphates,” “herbicides,” “insecticides,” “fungicides,” “bactericides,” “rodenticides,” “fumigants,” “attention deficit hyperactivity disorder,” and “ADHD”. The detailed search strategy for PubMed is shown in Table 1; similar search strategies will be used for the other databases. No language restrictions will be applied. The reference lists of the retrieved articles will be scanned to find if there was any additional literature.

**Table 1:** Search strategy used in electronic databases.

Number	Search terms
#1	Pesticide
#2	Herbicide or chlorophenoxy or dichlorprop or mecoprop or 2-methyl-4-chlorophenoxyacetic acid or 2,4-dichlorophenoxyacetic acid or 2,4,5-trichlorophenoxyacetic acid or dicamba or triazines or atrazine or amides or propanil or bipyridines or paraquat or diquat or thiocarbamates or S-ethyl-N,N-dipropylthiocarbamate or butylate or chloroacetanilides or alachlor or acetochlor or metolachlor or imidazolinones or imazethapyr or dinitroanilines or pendimethalin or phosphonoglycines or glyphosate
#3	Insecticide or organochlorines or dichlorodiphenylethanes or dichlorodiphenyldichloroethane or dichlorodiphenyltrichloroethane or dicofol or benzenes or lindane or hexachlorobenzene or chlorinated cyclohexanes or cyclodienes or aldrin or dieldrin or endosulfan or chlordane or heptachlor or toxaphene or chlordecone or mirex or organophosphates or chlorpyrifos or diazinon or fonofos or parathion or malathion or carbamates or carbaryl or aldicarb or aminocarb or pyrethroids or pyrethrins or permethrin or deltamethrin or cypermethrin or rotenone or microbiologicals or Bacillus thuringiensis
#4	Fungicide or dithiocarbamates or maneb or mancozeb or benzimidazoles or benomyl or captan or captafol or pentachlorophenol or iprodione or sulphur
#5	Bactericide or chlorine or chlorine-releasing agents or dichloronitrobenzene or triazine-S-triones
#6	Rodenticide or anticoagulants or bromadiolone or chlorophacinone or difethialone or diphacinone or brodifacoum or warfarinor or zinc phosphide or sodium fluoroacetate or bromethalin or cholecalciferol or strychnine
#7	Fumigant or methyl bromide or phosphine gas or magnesium phosphide or aluminium phosphide
#8	#1 OR #2 OR #3 OR #4 OR #5 OR #6 OR #7
#9	Attention Deficit Disorder with Hyperactivity OR ADDH OR Attention Deficit-Hyperactivity Disorder OR ADHD OR Attention Deficit Hyperactivity Disorder OR Attention Deficit Disorder OR Deficit Disorder OR Attention Deficit OR Hyperkinetic Syndrome OR Minimal Brain Dysfunction
#10	Pregnancy OR pregnant OR prenatal OR maternal
#11	#8 AND #9 AND #10

### 2.6 Study selection and data extraction

Two independent researchers will perform the study selection and data extraction. Any discrepancies will be resolved by discussion with an experienced third researcher. The EndNote reference manager will be applied to store and manage the tiles and abstracts of all of the studies identified by the literature search. Titles, abstracts, or full texts of the candidate studies will be scanned to identify eligible studies. Using a standard data collection form developed prior to data extraction, the following data from the included studies will be recorded: name of the first author, publication year, country, study design, sample size, ascertainment of ADHD, measurement of pesticide exposure, indicators for quality assessment, the most fully adjusted risk estimate, the corresponding 95% CI, and confounding factors.

### 2.7 Assessment of study quality and publication bias

The Newcastle-Ottawa Scales^[20]^ will be used to assess the methodological quality of cohort and case-control studies. This tool evaluates the selection of study groups (maximum of 4 stars), comparability of the study populations (maximum of 2 stars), and ascertainment of outcomes (for cohort studies) or exposure (for case-control studies) (maximum of 3 stars). A high score indicates a low risk of methodological quality. The scale of the Agency for Healthcare Research and Quality^[21]^ will be used to assess cross-sectional studies. The Begg test^[22]^ and Egger test^[23]^ will be used to detect potential publication bias.

### 2.8 Dealing with missing data

The corresponding author will be contacted by email to ask for original data if missing or unclear data is identified from the included studies. Otherwise, the existing data will be used for analysis.

### 2.9 Statistical analysis

STATA version 12.0 (Stata Corporation, College Station, TX) will be used to conduct the meta-analysis. The association between maternal exposure to pesticide with ADHD risk in offspring will be quantified using OR values and the corresponding 95% CIs. Between-study heterogeneity will be evaluated by the *I*^2^ statistic.^[24]^ The heterogeneity will be considered statistically insignificant if *I*^2^ ≤ 50%, then the fixed-effect model will be used to calculate pooled OR among the studies, otherwise, the random-effect model will be adopted.^[25]^ Subgroup analysis will be conducted according to study design, study location, sub-type of pesticides, ascertainment of ADHD, measurement of pesticide exposure, study quality, and adjustment for confounding factors. Sensitivity analysis will be performed by omitting the risk estimate of each study in turn to examine the robustness and stability of the pooled results.

## 3 Presenting and reporting the results

**Figure 1:**
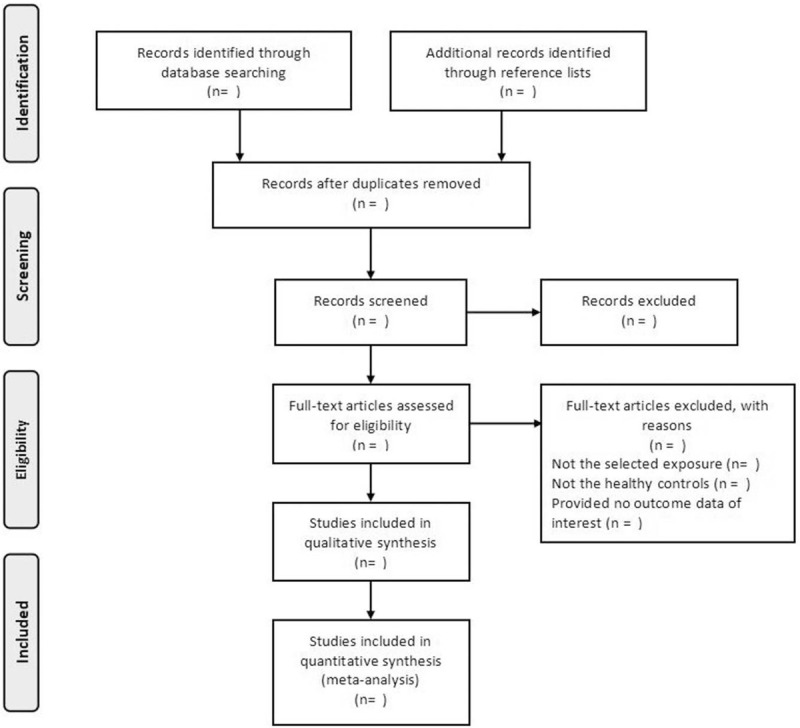
Flow chart of study selection process.

The selection process will be presented in a flow chart (Fig. 1). The characteristics of eligible studies, including the first author, publication year, country, study design, sample size, ascertainment of ADHD, measurement of pesticide exposure, adjusted RR and 95% CI, and confounding factors, will be displayed in a table. The Newcastle-Ottawa scale scores for included studies will be presented in a supplementary table. The results of meta-analysis, including the overall RR, RRs for subgroups, the corresponding 95% CIs, *I*^2^ statistic, and *P* value for heterogeneity will be summarized in another table. A forest plot will be presented in a figure to show the RRs for individual studies and the pooled RR. A separate table will be presented to list the eligible studies we could not obtain raw data from after contacting the corresponding authors.

## 4 Conclusion

In this systematic review and meta-analysis, we will assess the association between maternal exposure to pesticides and the risk of developing ADHD in offspring based on existing literature according to a pre-prepared protocol. The results might help to identify some types of pesticide that may contribute to the increased risk of ADHD. It will be of great practical and theoretical significance for the primary prevention of ADHD, focusing on prenatal interventions in the future.

## Author contributions

**Conceptualization:** Zhixian Zhu, Liwei Cheng.

**Data curation:** Zhixian Zhu, Huawei Tan.

**Formal analysis:** Ying Wang, Shiming Tang.

**Funding acquisition:** Zhixian Zhu, Cheng Liu, Liwei Cheng.

**Investigation:** Ying Wang, Shiming Tang, Huawei Tan.

**Methodology:** Zhixian Zhu, Cheng Liu.

**Project administration:** Liwei Cheng.

**Resources:** Zhixian Zhu, Cheng Liu, Liwei Cheng.

**Software:** Ying Wang, Shiming Tang.

**Supervision:** Zhixian Zhu, Liwei Cheng.

**Validation:** Huawei Tan.

**Visualization:** Zhixian Zhu, Ying Wang.

**Writing – original draft:** Zhixian Zhu, Liwei Cheng.

**Writing – review & editing:** Zhixian Zhu, Ying Wang, Shiming Tang, Huawei Tan, Cheng Liu, Liwei Cheng.
